# Governing the Global Antimicrobial Commons: Introduction to Special Issue

**DOI:** 10.1007/s10728-019-00388-4

**Published:** 2020-03-31

**Authors:** Steven J. Hoffman, Julian Savulescu, Alberto Giubilini, Claas Kirchhelle, Susan Rogers Van Katwyk, Isaac Weldon, Brooke Campus, Mark Harrison, Hannah Maslen, Angela McLean

**Affiliations:** 1 Global Strategy Lab, Dahdaleh Institute for Global Health Research, Faculty of Health and Osgoode Hall Law School, York University, 4700 Keele Street, Dahdaleh Building 2120, Toronto, ON M3J 1P3 Canada; 2 Department of Global Health and Population, Harvard T H Chan School of Public Health, Harvard University, Boston, MA USA; 3 Department of Health Research Methods, Evidence, and Impact and McMaster Health Forum, McMaster University, Hamilton, Canada; 4 School of Epidemiology and Public Health, Faculty of Medicine, University of Ottawa, Ottawa, Canada; 5 Oxford Martin School, University of Oxford, Oxford, UK; 6 Oxford Uehiro Centre for Practical Ethics, University of Oxford, Oxford, UK; 7 Wellcome Centre for Ethics and Humanities, University of Oxford, Oxford, UK; 8 Department of Politics, York University, Toronto, Canada; 9 Wellcome Unit for the History of Medicine, University of Oxford, Oxford, UK; 10 Department of Zoology, University of Oxford, Oxford, UK

**Keywords:** Antimicrobial resistance, International law, Global health policy, Collective action

## Abstract

Antimicrobial resistance is one of the greatest public health crises of our time. The natural biological process that causes microbes to become resistant to antimicrobial drugs presents a complex social challenge requiring more effective and sustainable management of the global antimicrobial commons—the common pool of effective antimicrobials. This special issue of Health Care Analysis explores the potential of two legal approaches—one long-term and one short-term—for managing the antimicrobial commons. The first article explores the lessons for antimicrobial resistance that can be learned from recent climate change agreements, and the second article explores how existing international laws can be adapted to better support global action in the short-term.

## Introduction

Antimicrobial resistance (AMR) is one of the greatest public health crises of our time [28]. AMR is an evolutionary process whereby microbes acquire resistance to the antimicrobial substances we have long depended upon to stop their spread, including antibiotics, antifungals, and antivirals. The likelihood of drug resistance increases every time microbes are exposed to antimicrobial substances, and 80 years of antimicrobial use in global medicine and agriculture has accelerated the development of AMR. Much is now at stake. If global efforts are not properly mobilized, we risk losing the ability to treat even the most basic of infections. Already, according to one estimate, more than 700,000 people die each year because of drug-resistant infections; this death toll is expected to climb to 10 million per year (more than today’s 9.6 million cancer deaths) by 2050 if no action is taken [14].

The biological phenomenon of AMR presents a social challenge requiring a more sustainable management of the global antimicrobial commons—a common resource with global population need. A global market failure has emerged wherein a negative demand-side externality incentivizes over-use because the full social cost of consumption is not borne by users. Uncertainty and low projected returns on investment have also disincentivized the development of new antimicrobials [10, 15, 17, 22]. Making matters worse, many people around the world still live and die without access to the life-saving antimicrobial drugs that they need. A delicate balancing act is required to address the urgent need to expand access to life-saving drugs, while simultaneously reducing the future risk of resistance [5]. Antimicrobials, however, are not exclusively used for human health; they are also widely used in veterinary and agricultural settings to treat infections, prevent illness, and improve food yields. Any large-scale efforts to address AMR will need to consider the health and economic implications of these wider uses across the human, animal, agriculture, and environmental sectors.

## AMR is a Series of Interlinked Challenges

Unlike many recent global infectious disease threats like HIV/AIDS, Zika, and Ebola, AMR is not caused by a single pathogen. Rather, AMR is a threat posed by a natural biological mechanism of action: microbes, including a range of disease-causing bacteria, viruses, fungi, and parasites, can evolve to become more resistant or resilient to particular antimicrobial agents [8]. Bacteria in particular have biological mechanisms for sharing genetic materials such that resistance to antibiotics can spread quickly, even beyond the original microbial species. Often a resistant pathogen can be treated using a different antimicrobial drug; over time, however, some pathogens acquire resistance to multiple antimicrobial drugs, as in the case of extensively drug-resistant tuberculosis (XDR-TB). More rarely—for the time being—some pathogens become resistant to all available treatments, as in the case of totally drug-resistant tuberculosis (TDR-TB) and some carbapenem-resistant Enterobacteriaceae.

**Fig. 1:**
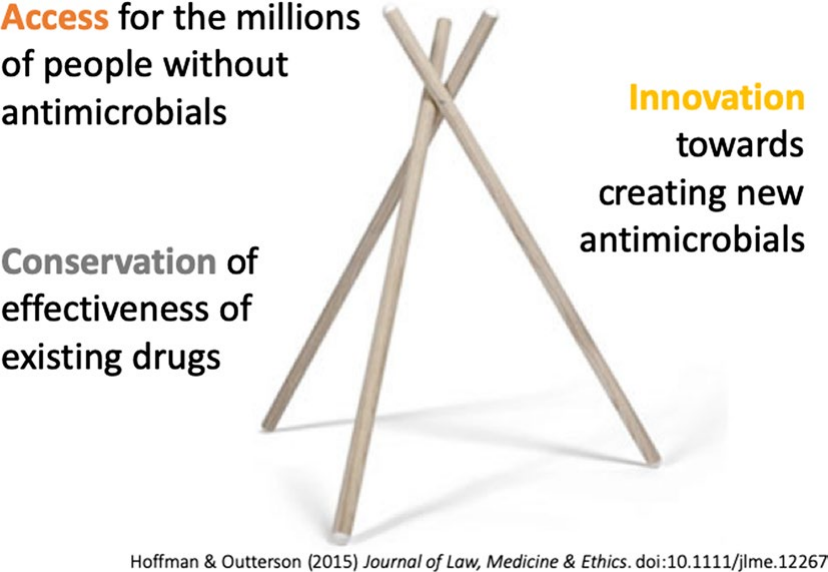
Policy tripod for addressing antimicrobial resistance

Broadly, AMR can be conceived of as three important and interlinked social challenges (Fig. 1) [4]. First is *access*: we need to ensure availability and affordability of antimicrobials for the millions of people each year who face life-threatening infections without them. Second is *conservation*: despite the need for access, we also need to ensure the sustainability of antimicrobials through disease prevention efforts, diagnostics, infection control, surveillance and appropriate prescribing. Third is *innovation*: we need to develop the next generation of antimicrobials to replace those that no longer work and to invent new vaccines, diagnostics, social responses, and infection control technologies to provide alternatives. These three interlinked challenges must be tackled simultaneously. Without conservation and innovation, universal access will increase resistance and deplete existing stocks of effective antimicrobials. Conservation, if pursued alone, will constrict the market for antimicrobials, restrict investment and innovation in the field, and further hinder access as well as driving resistance in low-income countries. Innovation without conservation mechanisms will quickly diminish the efficacy of new drugs and diminish the value of investments. And innovation without better access is inequitable: new life-saving technologies should not be denied to people based on geography, socio-economic status, or ability to pay.

Beyond these three social challenges, AMR poses specific technical challenges that underline the need for a multifaceted approach. First, there is a significant overall risk posed by the growing global reservoir of AMR-conferring genes, which circulate among bacterial organisms [9, 24]. Second, there are also particular “bug-drug” resistance pairings of urgent regional concern; resistance to anti-malarial drugs, for example, is of particular concern in malaria-endemic areas. Third, there are pathogens of particular global concern—often termed “critically important pathogens”—that show high or rapidly increasing levels of resistance to many available drugs. The World Health Organization (WHO) has published a list of these pathogens in the hopes of encouraging more research and development for new antimicrobials that specifically target them [25]. Fourth, there are “critically important antimicrobials,” key antimicrobial drugs that should be protected because they are in danger of completely losing their efficacy. In collaboration with the Food and Agriculture Organisation (FAO) and the World Organisation for Animal Health (OIE), WHO has published a list of the Highest Priority Critically Important Antimicrobials for human medicine [26], and developed “the AWaRe list,” a classification system grouping drugs into Access, Watch, and Reserve categories [21, 27]. The global community must be cautious, in light of these varied concerns, about taking overly simplistic approaches that cannot adequately address the social and technical complexity of AMR.

The global challenge posed by AMR is unique both in terms of its socio-technical complexity and longevity. Knowledge of AMR (first termed “drug fastness”) is already over a century old [23]; the first antimicrobial stewardship regimes date back to the 1940s, and awareness of AMR as an ecological problem defined by globally mobile genes and organisms arose during the 1960s [1, 3, 16]. From a biological perspective, past attempts to curb AMR have failed because of the historically uninterrupted rise of global antimicrobial use and subsequent proliferation of resistance-conferring genes [13]. From a political and social perspective, past attempts to tackle AMR have failed because stewardship regimes have been too narrow in national or sectoral scope, and policy responses at the international level have been fragmented [11, 12]. To date, the limited pool of “antimicrobial effectiveness” has not been effectively managed as a common-pool resource upon which the entire global community can sustainably depend, but rather has been left vulnerable to overuse and abuse by humans in the absence of regulation. Instead of international regulatory or policy approaches to manage the global AMR commons, past approaches to mitigating resistance have focused at the national and sub-national levels and have largely focused on changing the behavior of patients and prescribers [2, 19, 20]. Given that existing policies directed at changing individual patient and prescriber behaviour appear insufficient to control the acceleration of AMR, more institutionalized action seems necessary.

## Globally Governing AMR with International Law

Tackling AMR as a multi-sectoral issue depends on improving the global governance of the antimicrobial commons [2, 19]. Resistance genes spread easily between microbes, and the global movement of people and products enables microbes to transfer more easily among humans, animals, and the environment than ever before. For this reason, AMR presents a problem from which no country is immune and which no country can tackle alone. The clearest path forward is to significantly strengthen global coordination and collective action to sustainably manage antimicrobial effectiveness and contain the increasing threat posed by AMR.

In the following two papers, we argue that legal approaches represent the best path forward for achieving the necessary level of global coordination and collective action. International legal agreements represent the strongest formal mechanisms through which states can make commitments to each other [20], but require significant political mobilization and are rare in global health. If successful, though, an international legal agreement provides a regulatory framework that can bind countries together, provide accountability for turning commitments into action, and disincentivize parties from breaking their promises. AMR is one example of an issue where international regulations may accomplish what cannot be accomplished by individual countries or non-state actors acting alone.

## Conclusion

Ultimately, the effectiveness of any international legal approach to managing the global antimicrobial commons will depend on the willingness of countries to address AMR and to adopt sufficiently robust regulations that can mitigate the AMR threat [6, 7, 18]. We see two complementary international legal options going forward.

In the long-term, for global AMR efforts to be truly effective, the world needs an enduring international legal agreement governing this common-pool resource—a “grand bargain” tying together global efforts across sectors, countries, and time—on how we collectively manage the antimicrobial commons to balance access, conservation, and innovation. Grand bargains are complex agreements that fully address important common needs of their parties through a series of interconnected provisions. These may each be costly to an individual party but when implemented together will collectively benefit every party. A grand bargain on the global antimicrobial commons would strategically maximize the collective situation of countries facing AMR by ensuring sustained effectiveness of antimicrobials while ensuring each country’s infectious disease burden is meaningfully improved. While grand bargains are difficult to craft, they are helpful for promoting compliance with agreements over the longer-term because they are structured to ensure that each party is incentivized to uphold their obligations.

But AMR also requires action in the short-term. As a second complementary option, it could be helpful to identify “legal hooks” within existing international legal agreements where relevant AMR regulations can be developed or where small tweaks can be made to the agreement that would extend its scope to cover what is needed for containing AMR.

With either of these two legal approaches, developing a microbially effective, ethically fair, and politically feasible response to managing the global antimicrobial commons will require substantial effort to clearly define AMR priorities, set a global goal or target for AMR action, and develop an agreement that is sufficiently robust and widely implemented. The first article in this special issue describes the longer-term legal approach and explores the lessons for AMR that can be learned from recent climate change agreements. We recognize, however, that negotiating an international legal agreement is a massive undertaking and the threat posed by AMR necessitates substantial immediate action. Thus, the second article in this special issue explores how existing international laws can be adapted to better support global AMR action in the shorter-term. Such an approach can address pressing needs felt acutely today, including the need to strengthen AMR surveillance, fund the development of novel antimicrobials, and reduce the use of critically important antimicrobials in agriculture.
